# Supplementary material for: Impact of Hip Exercises on Postural Stability and Function in Patients with Chronic Lower Back Pain
Source: Diagnostics (Basel). 2025 May 13;15(10):1229. doi: 10.3390/diagnostics15101229 (PMC12110692; doi:10.3390/diagnostics15101229)
Supplement: Supplementary file 1 [file diagnostics-15-01229-s001.zip › diagnostics-3578671-supplementary.pdf]

Supplemental Table S1

Spinal stabilization exercises			
Exercise	Descriptions	Sets, reps, and resting time	Image
Side-bridge	Lie on your side with your knees bent, resting on your forearm and outer thigh. Slowly lift your hips off the ground, creating a straight line from your head to your heels. Hold the position for a few seconds, and then slowly lower your hips back down.	Two sets<br>10–15 reps<br>1–2 min rest<br>between sets	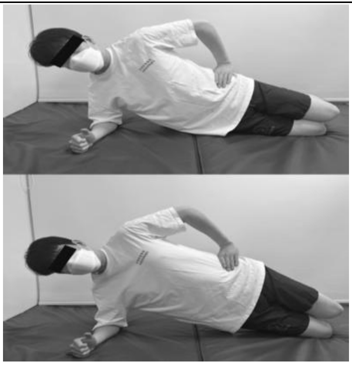
Bird-dog	Get on quaripedal position with your hands under shoulders and knees under hips. Extend one arm forward and the opposite leg backward simultaneously, creating a straight line from hand to foot. Hold this position for a few seconds, then slowly return to the starting position.	Two sets<br>10–15 reps<br>1–2 min rest<br>between sets	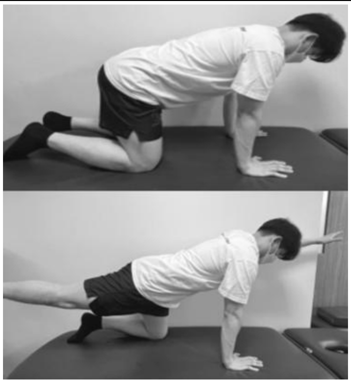
Pallof press	Tie elastic band on vertical bar. Stand on your feet and keep approximately 1 m from the bar. Grab the band with both hands and position it in front of your xiphoid process. Then slowly press both hands forward and backward while keep the resistance of the band.	Two sets<br>10–15 reps<br>1–2 min rest<br>between sets	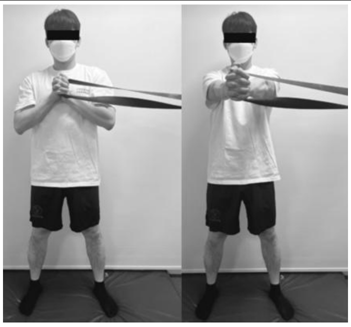

Hip exercises			
Exercise	Descriptions	Sets, reps, and resting time	Image

Supine bridge	Lie on your back with your knees bent, feet flat on the floor, and arms resting at your sides. Then push through your heels and lift your hips off the ground until your body forms a straight line from your shoulders to your knees. Squeeze your glutes at the top of the movement and slowly lower your hips back to the starting position.	Two sets<br>10–15 reps<br>1–2 min rest between sets	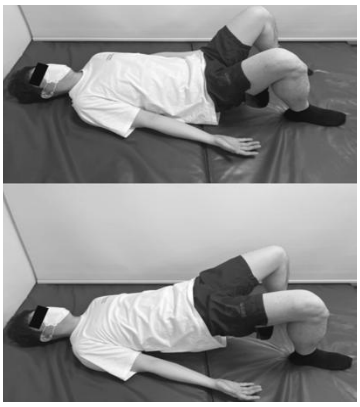
Supine bridge with single leg	Lie on your back with your knees bent, feet flat on the floor, and arms resting at your sides. Then push through your heels and lift your hips off the ground until your body forms a straight line from your shoulders to your knees. Squeeze your glutes at the top of the movement and lift non-working leg and extend it. After a short pause, slowly lower your hips back to the starting position.	Two sets<br>10–15 reps<br>1–2 min rest between sets	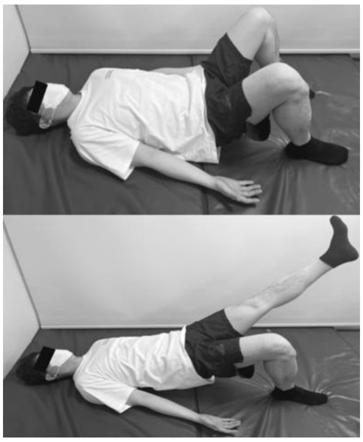
Clamshell	Lie on your side and loop both knee with elastic band. Make sure that your hips and knees are stacked on top of each other with 45° flexion. Keeping your heels together, lift your top knee upwards, aiming for a comfortable range of motion without rotating your low back. Hold the position for a few seconds on top, then slowly lower your top knee back to the starting position.	Two sets<br>10–15 reps<br>1–2 min rest between sets	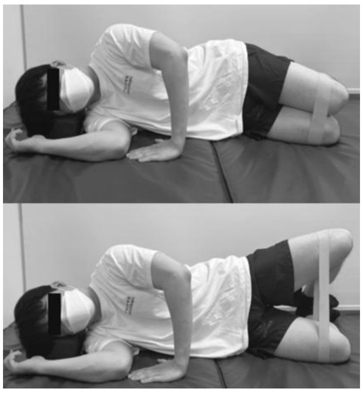

Hip abduction in single-leg standing with support	Loop a resistance band around your ankles. Stand on a non-working leg, and try to keep balance using supporting object. Working leg should be straight out to the side. Slowly lift working leg outward and slowly lower the leg back to the starting position.	Two sets<br>10–15 reps<br>1–2 min rest between sets	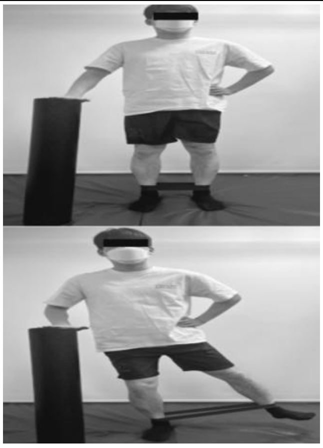
Hip abduction in single-leg standing without support	Loop the resistance band around your ankles. Stand on a non-working leg, keeping it slightly bent for stability. Working leg should be straight out to the side. Slowly lift working leg outward and slowly lower the leg back to the starting position.	Two sets<br>10–15 reps<br>1–2 min rest between sets	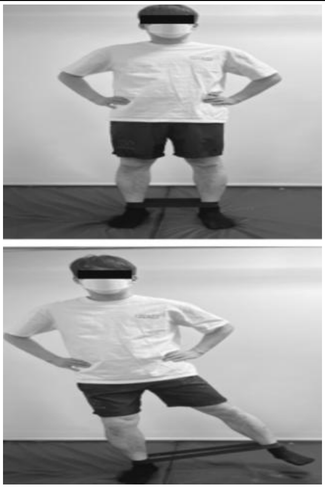
